# Extracranial Effects of Traumatic Brain Injury: A Narrative Review

**DOI:** 10.3390/clinpract15030047

**Published:** 2025-02-25

**Authors:** Nathan K. Evanson, Pratyusha Veldhi, Caitlyn Scherpenberg, John M. Riccobono, Haitham Eid, Jennifer L. McGuire

**Affiliations:** 1 Division of Pediatric Rehabilitation Medicine, Cincinnati Children’s Hospital Medical Center, Cincinnati, OH 45229, USA; 2 Department of Pediatrics, University of Cincinnati, Cincinnati, OH 45267, USA; 3 Department of Neurology and Rehabilitation Medicine, University of Cincinnati, Cincinnati, OH 45267, USA; 4 Kentucky College of Osteopathic Medicine, University of Pikeville, Pikeville, KY 41501, USA; 5 Medical Sciences Program, University of Cincinnati, Cincinnati, OH 45267, USA; 6 Department of Neurosurgery, University of Cincinnati, Cincinnati, OH 45267, USA

**Keywords:** traumatic brain injury, extracranial, cardiovascular, autonomic, renal, pulmonary, immune system, hemostasis, gastrointestinal, treatment

## Abstract

Background: Traumatic brain injury (TBI) is often associated with other injuries and comorbidities. However, even isolated TBI directly leads to dysfunction in multiple body systems outside the central nervous system. These extracranial effects of TBI target systems including the autonomic nervous, cardiovascular, renal, pulmonary, immune, gastrointestinal, and hemostasis systems, as well as causing significant alteration to systemic metabolism. Aim: This review is intended to outline the effects of TBI on other body systems, and place these in context with treatment considerations for these patients. Significance: Systemic effects of TBI have implications for acute and critical care management of patients with TBI, including pharmacologic treatment. They also affect treatment decisions in chronic TBI care, as well as TBI-unrelated routine medical care for patients with chronic TBI. In addition, extracranial effects of TBI should be considered in research settings. Conclusions: It is important for clinicians and researchers to be aware of these extracranial effects, and consider their effects on pathology, treatment decisions, and interpretation of research findings.

## 1. Introduction

Traumatic brain injury (TBI) is a major health concern, with at least 2.8 million cases occurring in the United States [1] and an estimated 69 million in the world [2] every year. In the United States, TBI is estimated to cost at least 60 billion USD per year in direct medical costs and lost productivity [3]. TBI also often occurs in the context of injury to other parts of the body sustained in the same traumatic event [4]. Comorbidities in other organ systems can have significant effects on the outcomes of acute care of TBI, whether these comorbidities are due to polytrauma occurring with TBI [5], or are due to pre-existing morbidity [6].

In addition to TBI-associated polytrauma and co-morbidities, TBI can also directly cause manifestations in systems outside the central nervous system. Such effects include changes in sympathetic and hormonal stress responses, impairments in organ systems, such as the cardiac, pulmonary, and renal systems, and changes in metabolism. Such TBI-induced extracranial effects are important to consider in outcomes and other pathologies that occur in the context of head trauma [7]. In addition, it can be important to consider these effects with respect to pharmacologic and other medical treatment of patients with traumatic brain injury.

**Figure 1:**
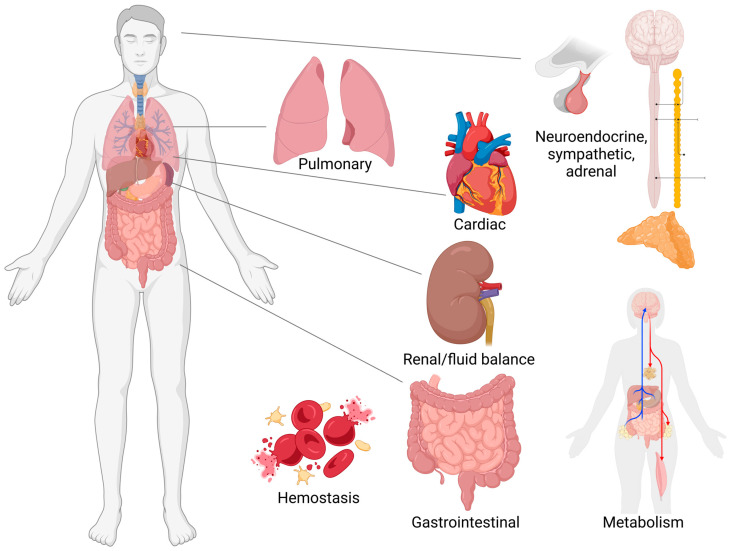
Overview of other systems affected by TBI. Created in BioRender. Evanson, N. (2025) https://BioRender.com/w64t126.

The purpose of this work is to review effects in other body systems that are induced by TBI (an overview of systems discussed is found in Figure 1). These systems are critical to consider, especially in the treatment of chronic TBI, because TBI is associated with earlier long-term mortality [8]. Although the cause of this decreased survival is not always clearly evident, it is likely at least in part due to the effects of TBI on other body systems, such as the cardiovascular system. Thus, consideration of extracranial effects of TBI can guide more complete treatment of chronic TBI patients. We note that, although the time frames of TBI (acute vs. chronic) have not always been consistently defined in the literature, the acute phase is commonly accepted to be the first week after injury, while chronic injury refers to more than 3 months after injury [9].

## 2. Autonomic and Adrenal Medullary

Many of the extracranial effects of TBI are driven or influenced by changes in the acute “fight-or-flight” responses of the sympathetic nervous system and adrenal medullary hormone changes (i.e., epinephrine and norepinephrine). Immediately after TBI, there is a significant increase of epinephrine and norepinephrine from the adrenal medulla, up to 500 times baseline for epinephrine and 100 times baseline for norepinephrine [10]. This abrupt surge of catecholamines is associated with paroxysmal sympathetic hyperactivity (PSH), seen most commonly with severe TBI [11]. PSH results in spikes of blood pressure, heart rate, blood pressure, and body temperature [12]. It is thought to be driven by reduction in descending inhibitory inputs to sympathetic nervous system centers [11]. Evidence that sympathetic hyperactivity involves increased catecholamines is also found in treatment studies. Treatment with beta-blockers, such as propranolol, is likely to be safe [13], may reduce mortality [13], and improves biomarkers of cardiovascular dysfunction and systemic inflammation [14]. However, efficacy for improving long-term outcomes is still sparse [15,16].

In addition to PSH, TBI frequently results in other types of autonomic dysregulation [17]. Even in milder TBI, such as in concussion, there is evidence of autonomic nervous system dysregulation, such as reduced heart rate variability [18]. Autonomic dysfunction after TBI affects both the parasympathetic and sympathetic nervous systems and can persist chronically even with good functional recovery from TBI [17]. Autonomic dysregulation from TBI can lead to dysfunction in other organ systems, prominently including the cardiovascular and renal systems [19]. Thus, it is important to consider autonomic dysfunction when discussing extracranial effects of traumatic brain injury, as well as their treatment options.

## 3. Immune System and Inflammation

After TBI, the body undergoes a complex inflammatory response, which initially includes the activation of immune cells and the release of pro-inflammatory cytokines [20,21]. This immune activation is driven at least in part by release of debris from injured nervous tissue, such as damage associated molecular patterns [22], leading to activation of both innate and adaptive immune responses [23]. Although this immune response is a necessary and protective part of the response to injury [24], over-activation of the inflammatory response can lead to a systemic inflammatory response syndrome, which is associated with worse TBI outcomes [25]. Given such potentially deleterious effects of increased inflammation and immune reaction after TBI, inflammation has been considered as a potential treatment target in TBI. However, results of studies targeting the immune system have been mixed, and at this point immunosuppressive medications, like glucocorticoids, are not recommended in treating TBI [26].

In addition to its initial pro-inflammatory effects, TBI can also cause immunosuppression that can be long-lasting [20,27,28]. In part, this immunosuppression may be responsible for increased infection rates in patients with more severe TBI, including pneumonia [29,30]. Post-TBI immunosuppression is driven by multiple mechanisms, including loss of peripheral lymphocytes [31], hypothalamus-pituitary-adrenal (HPA) axis and sympathetic nervous system activation [20,31], and immune exhaustion due to chronic activation of the immune system [30]. This post-TBI immunosuppression may be an important check against autoimmune activation [32].

In spite of the potential protective effect of immunosuppression after TBI, acquired brain injury, including TBI, does lead to autoimmune responses. It has been posited that autoimmunity leads to, for example, hyper-phosphorylated tau, and may cause significant long-term pathology in TBI [33]. TBI is known to cause increased autoantibodies against brain proteins in clinical populations [34]. In addition, TBI can increase the risk of autoimmune diseases, such as multiple sclerosis [35] or autoimmune hypopituitarism [36]. Overall, TBI disrupts normal regulation of immune cells, exacerbates pre-existing autoimmune conditions, and potentially induces new autoimmune reactions, complicating the recovery process and long-term outcomes.

## 4. Gastrointestinal and Brain-Gut Axis

It has long been recognized that patients with severe TBI have a high incidence of feeding intolerance [37] and delayed gastric emptying [38]. Patients with chronic TBI are also three times more likely to die from digestive disorders than age-matched individuals [39]. These issues are at least partially due to impaired gastrointestinal (GI) function [40]. This is likely to be largely due to disruption of the brain-gut axis, which is a bi-directional communication network between the brain and GI tract [41,42]. This communication is mediated by multiple mechanisms, including the autonomic nervous system and immune system, as well as blood transmission of compounds absorbed from the GI tract [43].

Multiple pathophysiological mechanisms contribute to GI dysfunction and disruption of the brain-gut axis, including changes in gut motility, barrier function, and dysbiosis [44]. In animal models, TBI leads to decreased intestinal contractility and increased transit time [45,46]. This dysfunction is accompanied by increased inflammatory cytokine levels and intestinal permeability [47]. TBI induces rapid structural changes in the intestinal mucosa [48], including reduced expression of tight-junction proteins [49]. These changes lead to impaired GI barrier function and increased gut permeability [43].

TBI also leads to bacterial dysbiosis and increased intestinal inflammation [46]. This is associated with rapid and substantial changes in the gastrointestinal microbiome composition [43], detectable within 2 h of injury [50]. These microbiome shifts are characterized by decreased bacterial diversity, generally leading to reduced beneficial bacteria and increases in potentially pathogenic bacteria [42]. Dysbiosis is likely at least a partial driver of the intestinal inflammation seen after TBI [42]. In association with increased permeability, dysbiosis leads to pro-inflammatory compounds being transmitted through the blood, back to the brain and thus driving neuro-inflammation and likely increasing secondary injury [43]. Because of the involvement of brain-gut axis disruption in TBI pathology, treatment approaches focused on GI disruption have been proposed for TBI patients [42].

## 5. Cardiovascular

Cardiac arrest is a significant complication of trauma in general [51], and occurs in isolated TBI [52]. Cardiac arrest in acute isolated TBI can be related to TBI-induced apnea [53]. Neurogenic stunned myocardium is another potential cause of cardiac arrest in TBI [54], and is thought to be caused by elevated catecholamines, with likely roles of systemic inflammation and neuroendocrine changes [55]. Cardiac rhythm changes after TBI have been described [56], which may also explain TBI-associated arrest. Myocardial dysfunction with TBI has been reported as improving after evacuation of a large subdural hematoma [57], also consistent with a significant relationship between TBI and cardiac arrest/dysfunction. Importantly, TBI-associated acute cardiac dysfunction is often reversible, so appropriate supportive care is recommended [58].

Outside the acute post-traumatic period, there is an increased risk of developing cardiovascular disease in chronic TBI [59,60], which may be worse in patients with repeated TBI [61]. This increased risk also extends to cerebrovascular disease, including stroke [60,62]. Hypertension is one of the most common comorbidities to develop in the 10 years after TBI [63]. Acute hypertension in TBI patients is associated with increased mortality [64]. In chronic TBI, hypertension and other cardiovascular risks are also increased [65], suggesting that TBI patients merit closer following and treatment of chronic cardiovascular disease. Interestingly, medical treatment of cardiovascular disease may improve brain health; for example, treating TBI patients with propranolol (a non-specific beta adrenergic antagonist sometimes used as an antihypertensive, or for cardiac arrhythmias) decreases biomarkers of neuroinflammation [14].

## 6. Hemostasis

Trauma in general causes changes in blood coagulation, known as trauma-induced coagulopathy [66]. Specifically, TBI causes coagulopathy, including dysregulation of platelet aggregation and fibrin clot formation [67]. Clinically significant coagulopathy in isolated severe head injury occurs in at least 1/3 to 2/3 of patients [68,69]. Although most common in severe brain trauma, coagulopathies can be identified in milder injuries, with increasing TBI severity associated with more significant platelet dysfunction [70]. Post-TBI coagulopathy is associated with progressive hemorrhage [69] and, unsurprisingly, patients on anticoagulation prior to TBI have worse clinical outcomes, likely due to increased hemorrhage risk [71].

Post-TBI coagulopathy is caused by multiple processes. After traumatic injury, the brain releases micro-particles into the circulation, which induce hypercoagulability [72]. This hypercoagulable state rapidly transitions into coagulopathy due to coagulation factors being consumed in a state of disseminated intravascular coagulation [73]. TBI also leads to inhibition of platelets via arachidonic acid and adenosine diphosphate signaling pathways [70]. TBI-induced coagulopathy can be exacerbated by factors like hypothermia, acidosis, and hemodilution, which further impair clot formation [74]. This disturbance in hemostasis not only increases the risk of bleeding in the brain but also complicates treatment strategies, as clinicians must carefully manage anticoagulation while preventing further hemorrhage. Thus, understanding and addressing coagulopathy in TBI patients is critical to improving outcomes and minimizing secondary brain injury.

## 7. Renal and Fluid Balance

TBI has effects on fluid and electrolyte balance, at least partially through altering renal function. Acute kidney injury is commonly seen early after TBI and is associated with higher TBI mortality [75,76]. TBI-associated fluid and electrolyte imbalance includes alterations in sodium, potassium, magnesium, and calcium [77]. Sodium abnormalities are the most common such alteration [78], and include hyponatremia [79] and hypernatremia [80]. Hyponatremia, hypernatremia, and even high sodium variability are associated with increased hospital mortality from TBI [79,80,81]. As would be expected, electrolyte alterations after TBI are often associated with changed renal function [77]. In the case of sodium, imbalances are most often caused by diabetes insipidus (DI), the syndrome of inappropriate antidiuretic hormone (SIADH), or cerebral salt wasting (CSW), but can also be caused by adrenal insufficiency or changed levels of natriuretic peptides [78].

DI is characterized by insufficient production or response to antidiuretic hormone (ADH), leading to excessive urination and volume depletion [82]. After TBI, DI is associated with hypothalamic or pituitary injury and thus with decreased ADH secretion. This leads to decreased reabsorption of water in the distal nephron, large-volume dilute urine production, hypernatremia, and usually to increased thirst [82]. Most often, DI is transient after TBI, although cases of prolonged or permanent DI have been described [82], including cases with delayed onset after TBI [83]. SIADH, on the other hand, results in excessive ADH secretion, causing water retention and thus hyponatremia [78]. As with DI, it is usually transient, but persistent cases have been described [84]. CSW involves the loss of sodium and water due to impaired renal sodium retention, leading to hyponatremia and hypovolemia [78]. All three conditions can emerge after TBI due to damage to the hypothalamus, pituitary gland, or areas of the brain that regulate fluid/electrolyte balance, and cases have been reported of patients having more than one of these conditions concurrently after TBI [85,86,87]. These conditions require careful management, as the fluid and electrolyte disturbances can exacerbate TBI outcomes and affect overall recovery.

## 8. Pulmonary

TBI can significantly impact pulmonary function and physiology. Immediately after TBI, there can be suppression or alteration of breathing, termed impact brain apnea [53]. Because impact brain apnea is not a physical obstruction in the airway, airway management alone will not return normal respiratory function. Its consequences can range from temporary cognitive deficits to long-term or permanent neurological damage or lethal cardiac failure due to hypoxia [88]. Impact brain apnea is thought to be caused by the effects of impact injury on brainstem function, and the duration of apnea correlates with the intensity of the impact [53]. Interestingly, treatment with caffeine immediately after experimental TBI in rats reduces or prevents impact brain apnea, which may implicate TBI-associated adenosine release in the pathophysiology of this entity [89].

In addition to impact brain apnea, TBI alters long-term pulmonary physiology and can cause pulmonary injury [90,91]. After TBI, brainstem damage can lead to irregularities in ventilation patterns and oxygenation [90]. The physical sequelae of TBI, such as prolonged immobility and altered consciousness, can increase the risk of respiratory complications, like atelectasis, neurogenic pulmonary edema, acute respiratory distress syndrome, and ventilator-associated pneumonia [92]. In fact, up to 20% of TBI patients develop acute respiratory distress syndrome, which increases mortality and has implications for critical care treatment [93]. In addition, TBI causes pulmonary injury via neurogenic pulmonary edema, and indirectly through systemic pro-inflammatory effects of TBI, and potentially via alterations in the gut-lung axis caused by TBI [94,95]. In addition, lung function post-TBI affects TBI outcomes [96], and lung inflammation post-injury may lead to worse cognitive outcomes after TBI [97].

## 9. Metabolism

Acutely in severe injuries, TBI patients experience hypermetabolism and associated weight loss despite enteral nutrition [98,99]. Basal energy expenditures in hospitalized patients can be as much as 200% higher than that expected based on standard predictive equations [100]. TBI-associated hypermetabolism can last for weeks [99] and result in significant weight loss [98]. In the rehabilitation phase, this weight is most often regained, and in a significant minority of cases, weight and BMI increase beyond pre-morbid levels during the first 1–3 years post-injury [98,101,102].

A number of factors promote weight change after TBI. Changes in food intake may occur, either up or down, suggesting changes in body weight set points in some individuals [98]. As could be expected, changes in mobility and motor function strongly contribute to early weight gain and increased BMI [98,101,103]. Impaired neurocognitive function and increased food intake associated with loss of executive control is another significant predictor of weight gain and obesity in the first years after TBI [98,103]. Changes in weight regulation after injury may contribute to cardiovascular and metabolic co-morbidity after TBI and an increased risk of metabolic syndrome, including hypertension, hyperlipidemia, and obesity [61].

Hyperglycemia is a frequent complication of TBI, is linked to unfavorable outcomes, and can be caused by factors such as stress responses and inflammation [104,105,106]. TBI is also associated with increased insulin resistance [107], which further contributes to hyperglycemia. Stress induced hyperglycemia is part of the physiological stress response to TBI, initiated by activation of the HPA axis and the sympathetic autonomic nervous system. This activation increases blood levels of catecholamines, cortisol, glucagon, and growth hormone, which enhance glycogenolysis and hypermetabolism, resulting in excessive glucose production [106,108]. Similarly, the activation of the systemic inflammatory response syndrome ultimately leads to elevated blood glucose levels [106]. This may occur in part due to alterations of glucose transporter levels and insulin signaling triggered by inflammatory cytokine actions [109], or by activation of hypothalamus-pituitary-adrenal axis activity by inflammation [110]. Interestingly, stress-induced hyperglycemia is associated with higher mortality compared to both normoglycemic patients and those with diabetic hyperglycemia [111,112].

TBI also has significant systemic effects on lipid metabolism, particularly in the liver. This occurs via multiple mechanisms, including increased lipid droplet accumulation and altered expression of metabolic regulators [113], including increased activation of phospholipase A2 [114]. TBI also leads to alteration of ketone and fatty acid oxidation [115], including increased lipid peroxidation in both brain and peripheral samples [116]. Patients with TBI display lipidomic changes that are correlated with patterns of pathology as identified in imaging studies [117]. TBI-induced lipid metabolism changes may be mediated at least in part by changes in growth hormone signaling [113]. Growth hormone deficiency after TBI leads to increased risk of metabolic syndrome and dyslipidemia [118], consistent with the finding that loss of growth hormone signaling in the liver leads to altered intrahepatic lipid metabolism and increased hepatic steatosis [119]. Changes in lipid metabolism may also be influenced by changes in gut microbiota induced by TBI [43]; the altered microbiota produce lipids that are absorbed by the patient and thus alter their lipid milieu.

## 10. Neuroendocrine

TBI can lead to clinically significant neuroendocrine deficits after TBI [120]. Such deficits are reported in about 8–20% of subjects with moderate to severe TBI [121,122]; deficits have also been reported in mild TBI [123]. Derangement of both anterior and posterior pituitary function occurs after TBI [124]; posterior pituitary dysfunction was addressed in the renal and fluid balance section above. The anterior pituitary gland is responsible for regulating hormone systems including growth hormone, gonadotropins, prolactin, thyroid hormones, and the hypothalamus-pituitary-adrenal stress hormone axis [125]. For the most part, significant pituitary injury leads to deficiency of the affected hormone system [126]. Prolactin is an exception to this pattern, because prolactin release is under tonic inhibition by hypothalamic dopamine; loss of this dopaminergic signal leads to increased prolactin release [127]. In spite of this, prolactin deficiency has also been reported in TBI patients [123].

The most common pituitary deficiencies described after TBI are alteration of growth hormone [120,128] and gonadotropin [129] secretion. Because of this, screening for pituitary deficiency is recommended for TBI patients [130]. Importantly, the way that neuroendocrine screening is performed can have an effect on the sensitivity of testing for abnormalities. For example, using an expanded definition of central hypothyroidism led to increased diagnosis in a small study (~16%) [131]. Thyroid insufficiency at more chronic time periods is relatively rare after TBI [120,122], although it can be common during the critical illness phase after TBI [132]. Typically, clinically significant post-TBI thyroid insufficiency is due to central hypothyroidism, so it is recommended to screen both thyroid stimulating hormone and free thyroxine levels [121,133]. Also less common, but of significant clinical implications, is dysfunction in the HPA axis.

Early after TBI, cortisol levels are elevated in human subjects [134] and corticosterone and adrenocorticotropin are elevated in animal studies [135]. In subacute times, adrenal insufficiency due to pituitary injury is described following TBI, but is a relatively rare deficiency in clinical populations [129,133]. Human clinical studies often use morning cortisol level to screen for HPA axis deficiency, although this may be insufficient as significant HPA axis dysregulation can occur without changes in basal glucocorticoid levels (e.g., [136]), particularly considering that TBI can cause alterations in circadian release of cortisol [137]. In animal studies using more sensitive dynamic testing of HPA axis responses to stressors, HPA axis over-activity is described early after injury [138,139]. In the subacute to chronic phase after injury both under-activity [140,141], and over-activity [142] have been described in animal studies. Changes in dynamic HPA axis activation may be mediated by altered regulation of corticosteroid receptor expression [138] and/or by changes in negative feedback regulation of corticosteroid secretion [143,144,145]. In addition, inflammation and neuro-inflammation interact with HPA axis responses after TBI, likely modifying both the HPA axis response and injury severity [146]. HPA axis changes in chronic TBI have also been postulated to affect susceptibility to conditions like post-traumatic stress disorder [147], suggesting this system may be an important treatment target for both TBI and related conditions.

## 11. Discussion

TBI commonly occurs in the context of polytrauma, and thus often has significant extracranial co-morbidities due to injury to other parts of the body. However, even in isolated TBI, there are significant effects on other organ systems in the body, that are caused by the TBI per se. These extracranial effects need to be kept in mind for acute and chronic care of TBI patients, and for long-term routine medical care in patients who have a history of TBI. In addition, extracranial effects of TBI can have significant implications in research settings.

With regard to acute care, it is well understood that TBI results in alterations in autonomic nervous system tone, metabolism, and fluid/electrolyte balance. These effects, to a great extent, inform current guidelines for critical care treatment of patients with TBI [148,149]. In particular, these guidelines focus on maintaining perfusion pressure and brain oxygenation, which are influenced by cardiac and pulmonary physiological changes. In addition, acute TBI care involves careful monitoring and treatment of fluid and electrolyte imbalance, particularly sodium [77]. Medication treatment in the acute/critical care phase of TBI, therefore, needs to consider these extracranial effects of TBI.

In the chronic phase of TBI, many of the acute care considerations are resolved or less common. For example, long-term paroxysmal sympathetic hyperactivity and diabetes insipidus are uncommon (although not unheard of). However, chronic changes in neuroendocrine, cardiovascular, and metabolic systems are still significant issues for chronic TBI patients [59]. In addition, chronic challenges, such as fatigue, can be associated with these changes [150].

In addition to implications for TBI treatment per se, extracranial consequences of TBI can have significant effects on long-term routine medical care. Perhaps the most obvious of the effects reviewed above in this arena are chronic changes in the cardiovascular system and metabolic system. Due to the increased incidence of chronic cardiovascular disease, metabolic syndrome, and insulin resistance, clinicians should maintain a higher vigilance in monitoring for these chronic diseases in patients with history of TBI and perhaps consider a lower threshold for pharmacological intervention.

In research arenas, extracranial effects of TBI should be considered when appropriate. For example, we have argued in the case of traumatic optic neuropathy that systemic effects of TBI, such as in the immune system, have effects on the pathophysiology of the condition [7,151]. In addition, TBI is associated with increased incidence of chronic neurodegeneration [152,153], including diseases like Alzheimer’s Disease [154] and other dementias [65,155]. Because extracranial effects of TBI, such as metabolic, immunological, and cardiovascular changes may influence the development of such disorders [156,157,158], it may be critical to consider the effects on these body systems in investigations of chronic neurological conditions after TBI.

The current review reveals areas where further understanding is needed. Segregating acute from chronic systemic effects of TBI makes sense from a treatment perspective, because clinical treatment of TBI in acute stages differs from chronic treatment. With regard to extracranial TBI effects, many can be seen in both acute and chronic TBI. In other cases, the degree of segregation between acute and chronic phases is not well understood. For example, posterior pituitary dysfunction (DI, SIADH, CSW) is most commonly described in acute and subacute TBI, but it is also reported in chronic TBI patients. Because most studies in this area focus on acute post-TBI times, it may be that chronic (especially mild) fluid dysregulation after TBI is under-reported. Similarly, there is a lack of clarity on which systemic effects of TBI are caused directly by brain injury rather than indirectly, such as via autonomic dysregulation. Since the pathophysiological mechanisms of most of the effects we have described are incompletely to poorly understood, much future work is needed to clarify how TBI leads to extracranial effects, and which effects are most important to consider in acute vs. chronic phases.

We also find that, to a large extent, standardized evaluation recommendations and guidelines are lacking for systemic effects in chronic TBI, although acute care treatment guidelines do consider at least some systemic effects. For example, current guidelines from the Brain Trauma Foundation address topics such as hyperosmolar treatment, ventilation, and nutrition [148]. Understandably, early TBI treatment recommendations are focused largely on improving mortality and short-term outcomes, consistent with the clinical goals of acute TBI treatment. With regard to chronic TBI, much less information is available, partly as a result of much less research being done on chronic than on acute TBI [159]. The paucity of information on chronic TBI treatment also occurs because interventions for chronic TBI patients, such as physical/occupational therapy, are harder to rigorously study, and endpoints are more difficult to assess than mortality scores. Future work should examine the importance of systemic effects of TBI to survival and functional recovery, especially at chronic time points. In particular, treatments that improve chronic disability due to TBI would be of great importance. Future work will also likely need to consider the complex, multi-systemic nature of TBI-induced deficits. Thus, we call for future studies to include assessment of multiple body systems and the use of diagnostic and treatment modalities to improve assessment and treatment in chronic TBI.

In conclusion, TBI per se has widespread effects on the body. Extracranial effects need to be considered in acute and chronic TBI treatment, in routine clinical care provided to patients with chronic TBI, and in research paradigms involving TBI and related conditions. Attention to these extracranial effects will aid in medication and other treatment as well as in research.

## Data Availability

No new data were created or analyzed in this study. Data sharing is not applicable to this article.

## Abbreviations

The following abbreviations are used in this manuscript:

ADH	Antidiuretic Hormone
CSW	Cerebral Salt Wasting
DI	Diabetes Insipidus
GI	Gastrointestinal
HPA	Hypothalamus-Pituitary-Adrenal
PSH	Paroxysmal Sympathetic Hyperactivity
SIADH	Syndrome of Inappropriate Anti-Diuretic Hormone secretion
TBI	Traumatic Brain Injury
